# The IL-15 / sIL-15Rα complex modulates immunity without effect on asthma features in mouse

**DOI:** 10.1186/s12931-020-1301-x

**Published:** 2020-01-29

**Authors:** Antoine Moui, Martin Klein, Dorian Hassoun, Eléonore Dijoux, Marie-Aude Cheminant, Antoine Magnan, Grégory Bouchaud

**Affiliations:** 1 L’institut du thorax, Inserm, CNRS, Université́ de Nantes, Nantes, France; 2 L’institut du thorax, CHU de Nantes, service de pneumologie, Nantes, France; 3 INRA, UR1268 BIA, rue de la Géraudière, F-44316 Nantes, France

**Keywords:** Interleukin-15, Interleukin-15 complex, Asthma, Allergy

## Abstract

**Background:**

Interleukin 15 (IL-15) is a growth and modulating factor for B, T lymphocytes and natural killer cells (NK). Its action on innate and adaptive immunity is modulated by its alpha chain receptor (IL-15Rα). The IL-15/sIL-15Rα complex (IL-15Cx) increases the bioavailability and activity of the cytokine in vivo. IL-15Cx has been used in diseases to dampen IL-15 inflammation by the use of soluble IL-15Ralpha specificity. Here, we aim to evaluate the interest of IL-15Cx in a mouse model of asthma.

**Methods:**

Using a mouse model of asthma consisting in percutaneous sensitization and intranasal challenge with total house dust mite extract, we evaluated the effect of IL-15Cx injected intraperitoneally four times after a first nasal challenge. Respiratory function was assessed by the technique of forced oscillations (Flexivent®). The effect on bronchial remodeling was evaluated by lung histology. The inflammatory status was analyzed by flow cytometry.

**Results:**

We observed that the IL-15Cx modulates lung and systemic inflammation by increasing NK cells, CD8^+^ memory T cells and regulatory cells. However, IL-15Cx displays no effect on bronchial hyperreactivity, bronchial remodeling nor cellular bronchial infiltrate, but limits the secretion of bronchial mucus and modulates only inflammatory response in a HDM-allergic asthma murine model.

**Conclusions:**

IL-15Cx has a limited effect on immune response in asthma and has no effect on lung function in mice. Thus, it limits its therapeutic potential but might suggest a combinatory potential with other therapeutics.

## Background

Interleukin 15 is a pleiotropic cytokine of the family of cytokines with 4 α-helices also including members such as IL-2, IL-3, IL-4, IL-6 and IL-21. It was discovered in 1994 as a cytokine inducing the proliferation of T cells independently of IL-2 but thanks to the β receptor of IL-2 (IL-2Rβ) [1]. It has similar functionalities to IL-2, with which it shares common receptor chains, IL-2 / 15Rβ (CD122) and γc (CD132) [2]. Cytokine specificity is conferred by the presence of their respective α-receptor chains, IL-15Rα (CD215) and IL-2Rα (CD25). IL-15Rα has a high affinity for IL-15 (kD ~ 10^− 11^ M) independently of the β and γc chain [3], and its mRNA is found in many tissues. Kennedy et al. determined the cellular response to IL-15 through the use of IL-15 and IL-15Rα deficient mice. Indeed, these mice had a deficiency in Natural Killer (NK) cells, Natural Killer T (NKT) cells, γδ T lymphocytes, naive and memory CD8^+^ T lymphocytes [4], demonstrating a role in innate and adaptive immunity. Furthermore, the main targets of IL-15 in vivo seem to be NK cells and CD8^+^ T lymphocytes since the administration of recombinant IL-15 to mice causes a rapid increase of these cells [5]. Its role in the proliferation and survival of memory CD8+ T cells is also well established [6]. IL-15 is synthesized in small amounts mainly by antigen presenting cells such as dendritic cells, monocytes and macrophages. When the IL-15 / IL-15Rα complex (IL-15Cx) is routed to the surface of these cells, it can stimulate NK cells and CD8^+^ T cells via their IL-2Rβ and γc receptor, thus allowing a functional response to IL-15. This mechanism of action of IL-15 in vivo is called trans-presentation [7, 8]. In 2004, it was shown that a soluble form of human IL-15Rα could also be released naturally from cells bearing this receptor, through an excretion process involving matrix metalloproteinases [9]. This soluble IL-15Rα receptor was able to bind to IL-15 with high affinity and effectively blocked IL-15 dependent proliferation of cells through the IL-15Rα/β/γ high affinity heterotrimeric signaling complex. In addition, the complex formed by IL-15 and the soluble IL-15Rα (IL-15Cx) is able to improve the binding as well as the bioactivity of IL-15 via the IL-15Rβ/γ intermediate affinity heterodimeric receptor [10]. These divergent results led to study the IL-15Cx in vivo in mouse models. Biological activity of IL-15 is improved after interaction with recombinant soluble IL-15Rα [11] and injection of the complex rapidly induced proliferation of memory CD8^+^ T cells and NK cells. These results could be explained by a conformational change of IL-15 after interaction with soluble IL-15Rα during IL-15Cx formation which potentiates the recognition of IL-15 by the βγc receptor on T lymphocytes, via a 13-amino acid (aa) peptide present on the encoded ectodomain by exon 3 [12]. The administration of an IL-15Cx will have a modulatory effect on the activity of the cytokine by allowing fixation only to cells expressing the dimeric βγc receptor. Another advantage of this complex is that it increases the biological activity of IL-15 by 50-fold and its half-life by 20-fold [13], regardless of the route of administration. The therapeutic utility was also tested by the same team by demonstrating that complexed IL-15 compared to IL-15 alone, significantly reduced tumor burden in a B16 melanoma model. The pulmonary inflammatory modulation of the IL-15Cx is not well documented and could be interesting for inflammatory diseases. Asthma is a common pathology with a significant inflammatory component, conducive to the study of the complex. IL-15 seems to have a role in the pathophysiology of allergic asthma. Recently, *Venkateshaiah* et al. showed that IL-15 deficiency increased airway resistance and decreased compliance in a mouse model of asthma [14]. Furthermore, administration of recombinant IL-15 decreased allergic airway obstruction. Overexpression of IL-15 increased interferon gamma and decreased pro-inflammatory Th2 cytokine levels as well as endothelial hyperplasia. Despite these interesting results, some points are still to be clarified. In fact, the model used was more a model of pulmonary inflammation than a model of allergy. The use of an allergic asthma model with a TH2 / TH17 inflammatory component approximating asthma in humans would better reflect the effect of IL-15 in asthma. In addition, using the IL-15Cx would prolong half-life and bioavailability of the cytokine, still in a human therapy projection. Here we investigated inflammatory modulation of IL-15Cx in steady state and its therapeutic potential in a mouse model of asthma.

## Methods

### Mouse model of acute allergic asthma to house dust mites

Female BALB/c mice 6 weeks old (JAX Mice BALB / cByJ, Charles River) were used for animal experimentation. As described previously [15], the model was constructed with two phases, a sensitization phase and a challenge phase (Fig. 4a). Before each allergenic exposure, mice were anesthetized by intraperitoneal injection (IP) of 100 μl of a mixture of ketamine (80 mg/kg) and xylazine (15 mg/kg). The allergen used was house dust mites (HDM) (Stallergenes, Antony, France) containing *Dermatophagoides pteronyssinus*. Five hundred micrograms of pure HDM diluted in dimethylsulfoxide (DMSO, Sigma-Aldrich, L’Isle d’Abeau Chesnes, France) were applied percutaneously to both sides of each ear. Four weekly applications over 3 weeks were performed (D0, D7, D14 and D21). For control mice, DMSO 70% diluted in isotonic saline (NaCl) was applied percutaneously. The challenge phase included two challenges 1 week apart (D27 and D34). Two hundred and fifty μg of HDM diluted in phosphate buffered saline (PBS) were inoculated intranasally using a micropipette. The control mice received only PBS. This protocol received a favorable opinion from the Pays de la Loire ethics committee under number 9456. The analyzes were performed the day after the second challenge (J35), after a sacrifice by injecting a lethal dose of EUTHASOL (150 mg/kg intraperitoneally).

### IL-15Cx

Human recombinant IL-15 (12.9 k Dalton, Peprotech France) and human IL-15 Rα-Fc (42.6 k Dalton R & D Systems) were reconstituted in PBS at concentrations of 10 μg/mL and 60 μg/mL respectively. The complex was prepared with a molar ratio of 1 to 1. Both components were mixed together for 20 min at room temperature before injection. Between the two challenge phases, control and asthmatic treated mice received four injections of 100 μL of the complex composed by 0.5 μg of rhIL-15 and 3 μg of rhIL-15Rα-Fc. The mice not treated with the complex received 100 μl of PBS intraperitoneally.

### Airway hyperresponsiveness measurements

Tracheotomy was performed surgically on anesthetized mice and a cannula was inserted. The mice were placed in the Finepoint RC system room (Data Sciences International, St Paul, Minnesota) and connected to a ventilator. Mechanical ventilation was started with a respiratory rate of 150/min and a tidal volume of 0.15 mL as previously described [16]. The mice were nebulized with NaCL (baseline reference data) then with methacholine at increasing doses (5, 10, 15 and 20 mg/L). Aerosols of 20 μL were generated by an ultrasonic nebulizer and aspirated through the chamber for 30 s. Resistance (cmH2O.sec/mL) was measured with an average of the values generated within 3 min after each nebulization. At the end of the protocol, the mice were sacrificed.

### Histology

For each condition, lungs were perfused with 4% paraformaldehyde, then removed and deposited in the same solution. The right and left lungs were separated and cut perpendicular to the insertion of the bronchus and then embedded in paraffin using an inclusion automaton. Histological sections of 7 μm thick were made using a microtome on the paraffin blocks. These sections were then stained with Hematoxylin & Eosin staining for morphological studies and calculation of a histological score. Micrographs of the stained sections were then taken under the microscope to allow analysis on the ImageJ software (Cambridge, UK). The histological score was calculated on 12 points, of which 8 points to evaluate the bronchial inflammation and 4 points for the bronchial remodeling. Schiff periodic acid staining (PAS) was also applied for each condition on different sections to evaluate bronchial mucus secretion score. This score was calculated by counting 5 bronchial sections per group, the total number of PAS positive cells (goblet cells) relative to the total number of epithelial cells of this bronchus. Scores were evaluated by blinded observers.

### Flow cytometry

After mice dissection, lungs, mediastinal lymph nodes and spleens were collected in 15 mL of RPMI, milled and filtered through 40 μm cell sieves. We also collected the bronchoalveolar lavages by intra-tracheal injection then aspiration of 1 ml of PBS. After centrifugation for 5 min at 260 relative centrifugal force (RCF), the lung pellet and spleen were suspended in Red Blood Cell lysis (RBC, Sigma) to destroy the red blood cells followed by an addition of 9 mL of FACS Buffer (1% 0.5 M EDTA pH 8, 5% FCS, 1X DPBS without Ca2^+^ without Mg2^+^, Gibco) to stop the action of RBC. The sample cells were then counted on a KOVA slide (California, USA). In parallel, each sample was deposited on a 96-well conical bottom plate. The cells were then centrifuged for 2 min at 310 RCF and then incubated for 20 min at 4 °C with the various antibodies in the presence of Fc blocker CD16/32 (BD Bioscience). For extracellular labeling each antibody was diluted in FACS Buffer: CCR3-AF647, CD3-PerCPCy5.5, Ly6C-PE, CD4-APC-Cy7, CD25-PE-Cy7, CD8-FITC, CD44-BV421, CD122-PE, CD11c-APC and MHC II-BV510 (Sony, UK), NKp46-AF700, DX5-PeCy7 and CXCR3-BV510 (Biosciences, Claix Bridge) and F4/80-FITC (Biolegend, San Diego, USA). After extracellular labeling, the samples were centrifuged and then resuspended in 200 μL of FACS Buffer. The cells were centrifuged for 2 min at 310 RCF at 4 °C and then permeabilized with a fixing/ permeabilization solution (BD Phosflow™, BD Biosciences) for 15 min at 4 °C in the dark. They were then incubated for 40 min at 4 °C with the FoxP3-AF647 antibody (Sony) in the permeabilization solution. Finally, we resuspended them in 200 μL of FACS Buffer. The cells were analyzed by flow cytometry on the Fortessa TM LSRII (BD Biosciences) and the data acquired on the FACS Diva 8 software (BD Biosciences). Data were analyzed on the Flowjo software (TreeStar, version 10.4).

### Statistical analysis

Statistical analysis to compare the different groups of mice were performed using the GraphPad Prism 7 software (version 7.0a; GraphPad Software, San Diego, California). The D’Agostino Pearson and Bartlett’s tests were performed to test the normality of the values and the difference of the variances respectively. Subsequently, the nonparametric Mann Whitney test was used to compare for non-normal-law variables and the parametric t-test for normal-law variables, as indicated in figure legend. Values are represented on figures with mean ± SEM (Standard Error of the Mean). A value of *p* < 0.05 was considered statistically significant.

## Results

### IL-15 complex modulates lung inflammation

**Fig. 1:**
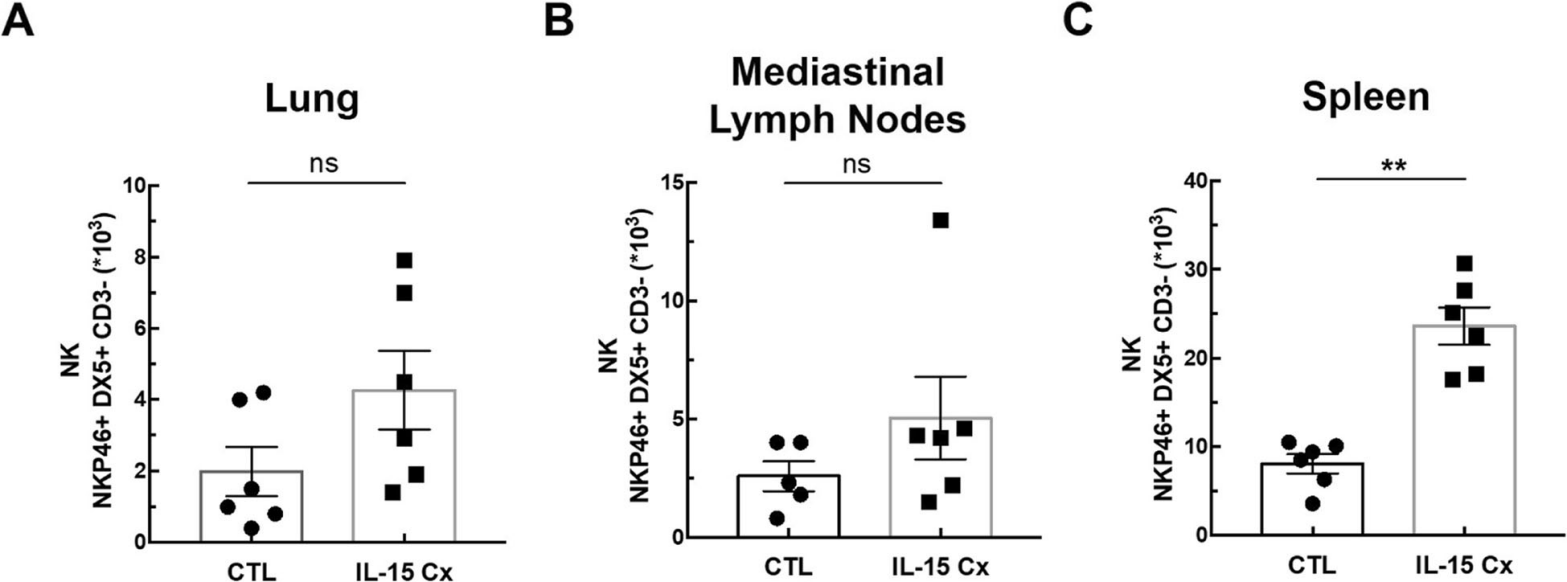
IL-15 complex has a systemic effect on NK cells, but also in the lungs. The numbers of NK cells (NKP46^+^, DX5^+^, CD3^−^) were respectively measured in (**a**) lungs, (**b**) mediastinal lymph nodes and (**c**) spleen by flow cytometry. The dissected lungs, mediastinal lymph nodes, and spleen from control mice (CTL) and mice treated by IL-15 complex (IL-15 Cx) were crushed, filtered, and then lysed by Red Blood Cell lysis (RBC) for lungs and spleen. The data are represented as mean ± SEM (between 5 and 6 mice per group); * *p* < 0.05, ** *p* < 0.01, using non parametric Mann-Whitney test. ns = not significant

**Fig. 2:**
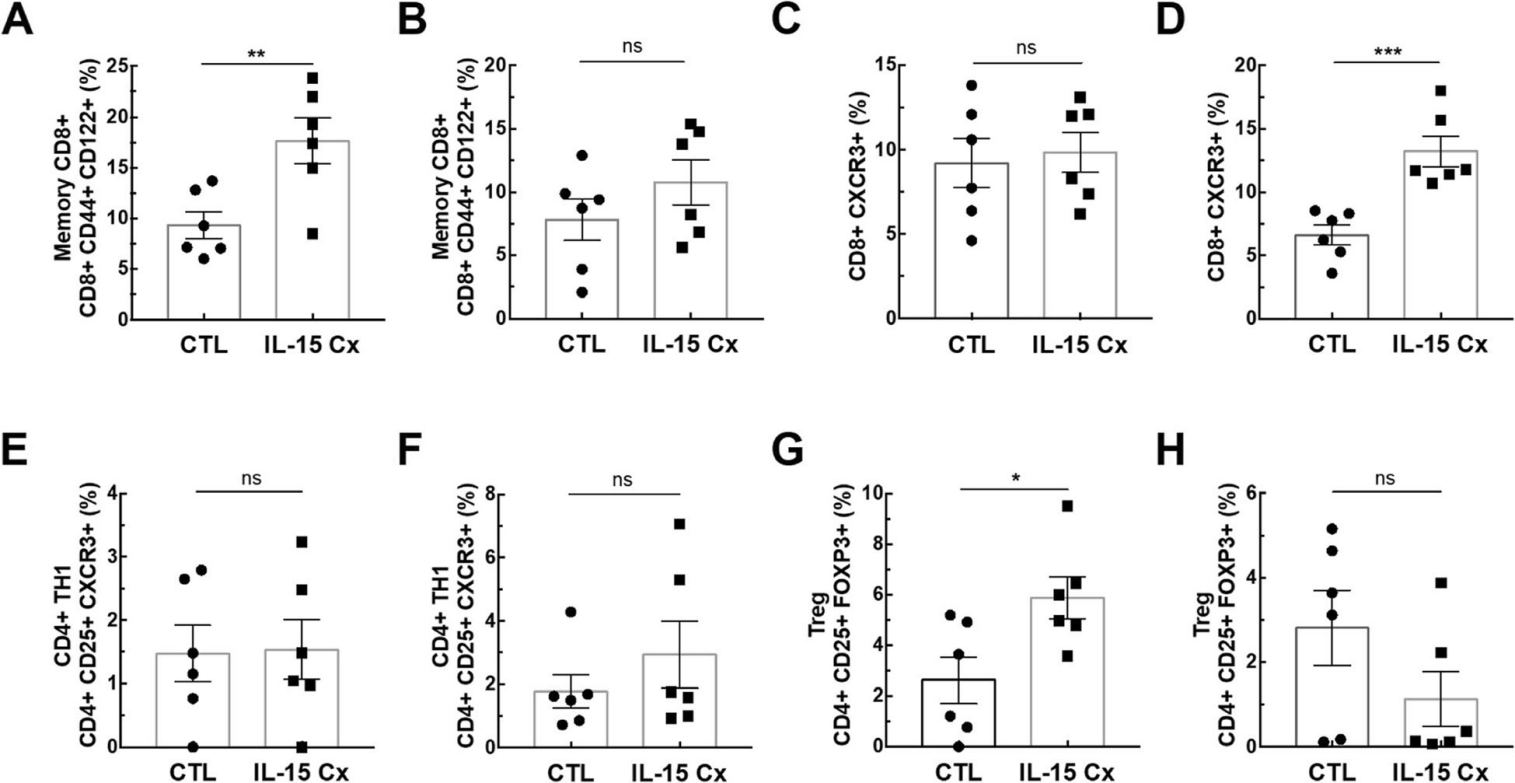
IL-15 complex modulates adaptative immunity. The percentage of (**a**, **b**) Memory CD8^+^ lymphocytes (CD8^+^, CD44^+^, CD122^+^), **(c**, **d)** CD8^+^ lymphocytes secreting interferon gamma (CD8^+^, CXCR3^+^), (**e**, **f**) CD4^+^ Th1 lymphocytes (CD4^+^, CD25^+^, CXCR3^+^) and (**g**, **h**) T regulatory lymphocytes (Treg) (CD4^+^, CD25^+^, FOXP3^+^) were measured in (**a**, **c**, **e**, **g**) lungs and **(b**, **d**, **f**, **h)** spleen. The dissected lungs and spleen from CTL and IL-15 Cx mice were crushed, filtered, and then lysed by RBC for lungs and spleen. The data are represented as mean ± SEM (between 5 and 6 mice per group); * *p* < 0.05, ** *p* < 0.01, *** *p* < 0,001 using parametric t-test. ns = not significant

**Fig. 3:**
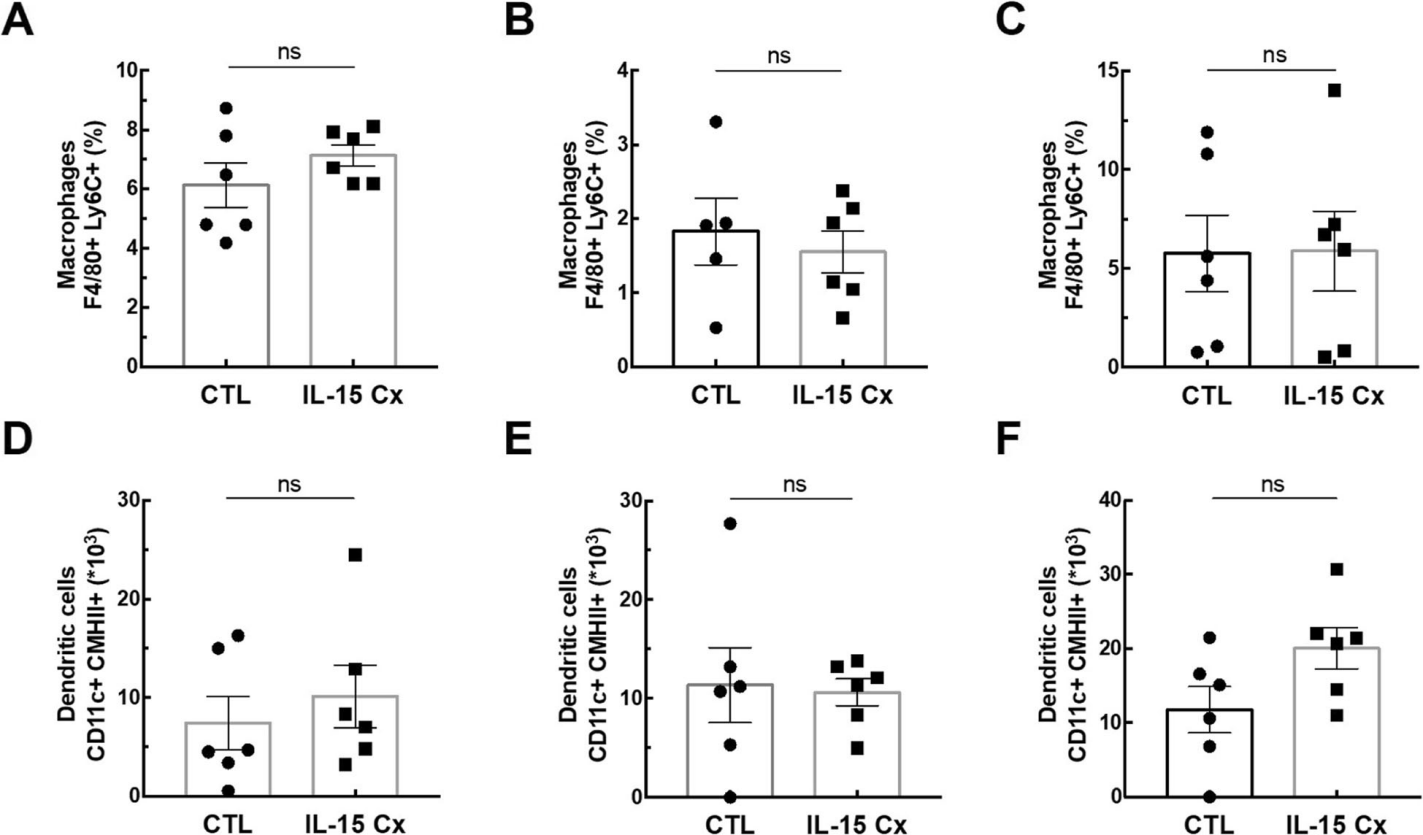
Interleukin 15 secreting cells are not modified by IL-15 complex. The percentage of macrophages (F4/80^+^, Ly6C^+^) and the number of dendritic cells (CD11c^+^, CMHII^+^) were measured respectively in (**a**, **d**) lungs, (**b**, **e**) mediastinal lymph nodes and (**c**, **f**) spleen by flow cytometry. The dissected lungs, mediastinal lymph nodes and spleen from CTL and IL-15 Cx mice were crushed, filtered, and then lysed by RBC for lungs and spleen. The data are represented as mean ± SEM (n between 5 and 6 mice per group); * *p* < 0.05 using parametric t-test. ns = not significant

In order to explore the potential of IL-15Cx on the lungs, we analyzed innate and adaptive responses in control mice (CTL) and to mice which received four injections of the IL-15 complex (IL-15Cx group) over 1 week. We first looked at NK cells (NKP46^+^, DX5^+^, CD3^−^), a population increased by injection of the complex in spleen but not in lungs and mediastinal lymph nodes **(**Fig. 1**)**. In the lungs, there was no statistically significant difference but a tendency to an increase of NK cells in IL-15Cx compared to CTL (4267 ± 1100 cells versus 1983 ± 685 respectively, *p* = 0,09) **(**Fig. 1a**)**. There was no evidence of a change in the number of NK cells in the mediastinal lymph nodes after injection of the IL-15Cx **(**Fig. 1b**)**. However, based on findings from past studies on IL-15Cx, we observed an increase in NK cells in IL-15Cx treated group compared to CTL in the spleen (23,630 ± 2120 cells versus 8060 ± 1080 respectively, *p* = 0,002) **(**Fig. 1c**)**. Given an almost significant difference on NK cells in the lung, we wanted to further explore the pulmonary and systemic inflammatory effects of the complex on other cell populations. To this end, we analyzed CD8 and CD4 lymphocytes subpopulations **(**Fig. 2). We observed an increase in CD8^+^ lymphocytes (CD8^+^, CD44^+^, CD122^+^) in lung after administration of the complex (9,3% versus 17,4% respectively, *p* = 0,009) **(**Fig. 2a), but not in the spleen **(**Fig. 2b). On the contrary, CD8^+^ lymphocytes expressing the CXCR3 receptor (CD8^+^, CXCR3^+^) were increased in the spleen (6,6% versus 13,22% respectively, *p* = 0,001) **(**Fig. 2d) but not in the lung **(**Fig. 2c). Moreover, we analyzed CD4^+^ T-helper cells in the lungs **(**Fig. 2e) and in the spleen (Fig. 2f) and no effect was observed. On the other hand, when we explored the effect of IL-15Cx on T regulatory lymphocytes (Treg) (CD4^+^, CD25^+^, FOXP3^+^), and found an increase of their percentage in the lungs (2,62% in CTL versus 5,88% in IL15Cx, p = 0,02) **(**Fig. 2g) but not in the spleen (2,81% versus 1,13% respectively, p = 0,15) **(**Fig. 2h). Altogether, IL-15Cx modulates adaptive immunity and may have an effect on innate cells. Among them, dendritic cells and macrophages are known to be partially dependent of IL-15 for their homeostasis. These cells secrete IL-15 and activate other cells by transpresentation through the IL-15Rα. Administration of the IL-15 complex containing the IL-15Rα should theoretically not activate these cells but we wanted to clarify this hypothesis in vivo. We performed flow cytometry on lungs **(**Fig. 3a and d), mediastinal lymph nodes **(**Fig. 3b and e) and spleen (Fig. 3c and f) in CTL and IL-15Cx group to analyze macrophages and dendritic cells respectively. We did not observe significant difference after administration of the IL-15Cx (Fig. 3) on both cell populations. These results are consistent with the mechanism of action of the complex and in particular the absence of activation of the cells expressing the soluble receptor alpha of IL-15.

### The IL-15 complex does not improve asthma features

**Fig. 4:**
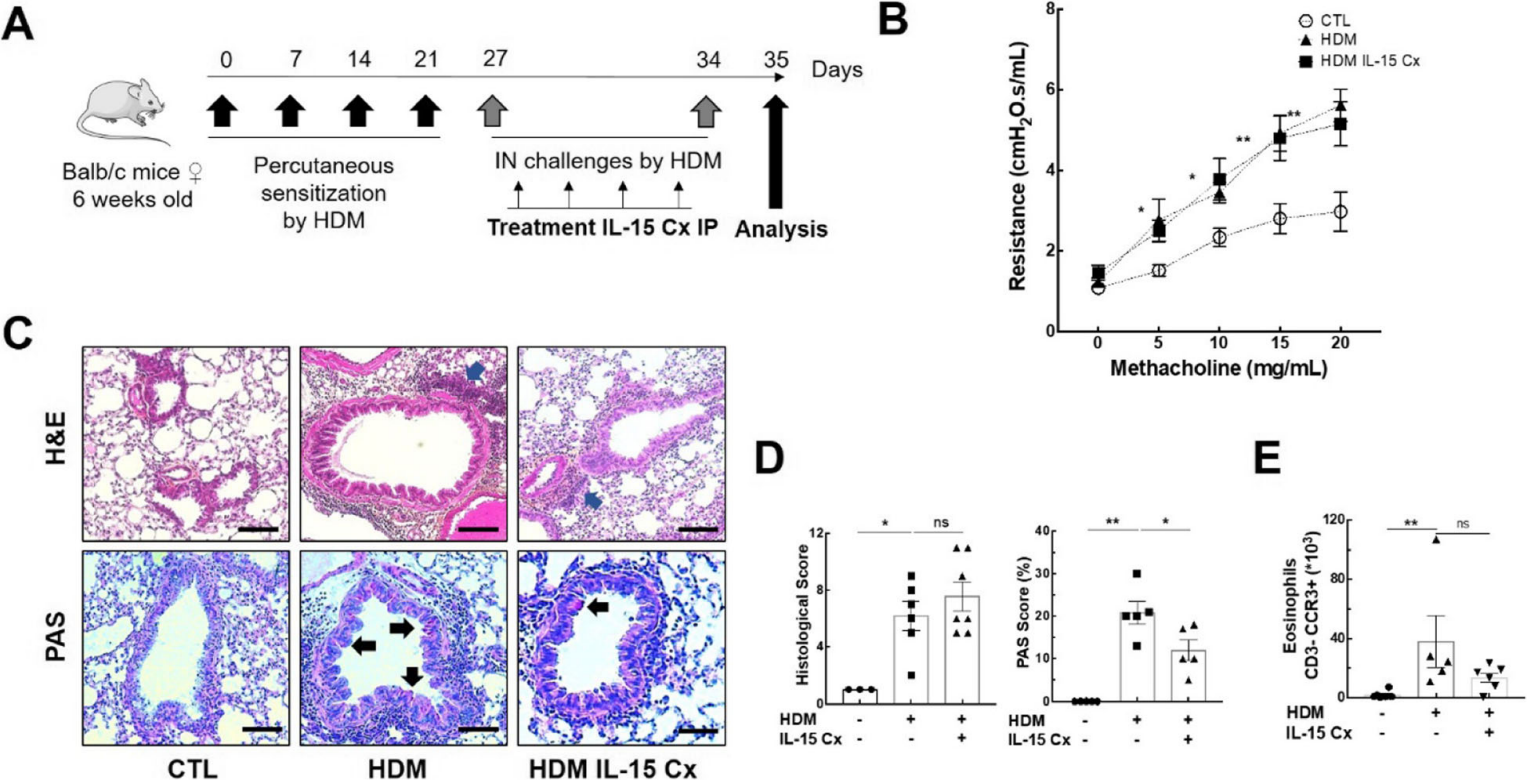
Asthma features are not improved by IL-15 complex. **a** Murine model of acute allergic asthma. Sensitization of Balb/c mice by four percutaneous applications of 500 μg of total mite extracts (HDM). For challenges, 250 μg of HDM were inoculated intranasally (IN). Four injections of the IL-15 complex and its soluble alpha receptor (IL-15 Cx) were administered intraperitoneally (IP) once daily between the two challenges. Analysis were made the day after the second challenge. **b** Airway resistance (RI; cmH2O/mL) in anesthetized CTL, asthmatic group (HDM) and asthmatic mice treated by IL-15Cx (HDM IL-15 Cx), in response to various concentrations (0, 5, 10, 15, 20 mg/mL) of methacholine were measured with Finepoint RC system. **c** Lungs were perfused with 4% paraformaldehyde solution and stained with hematoxylin and eosin (H&E) or periodic Schiff acid (PAS). Bronchial histology was performed for CTL, HDM and HDM IL-15 Cx group. Blue arrows: inflammatory peri-bronchovascular infiltrate. Black arrows: goblet cells colored with PAS. Scale = 100 μm (**d**) Histological sections were scored on 12 points; 4 points are devoted to morphological alteration and 8 points to inflammation (*n* = 3 to 7 mice per group); The PAS score was calculated on 5 bronchial sections per group, as the number of positive PAS cells (black arrows) reported to the total number of epithelial cells of this bronchus. **e** Eosinophils (CD3-, CCR3+) were measured in bronchoalveolar lavage fluid by flow cytometry. The cells were collected by washing the lung with 1 ml of PBS after tracheostomy.* *p* < 0.05, ** *p* < 0.01 using Mann-Whitney test. ns = not significant

**Fig. 5:**
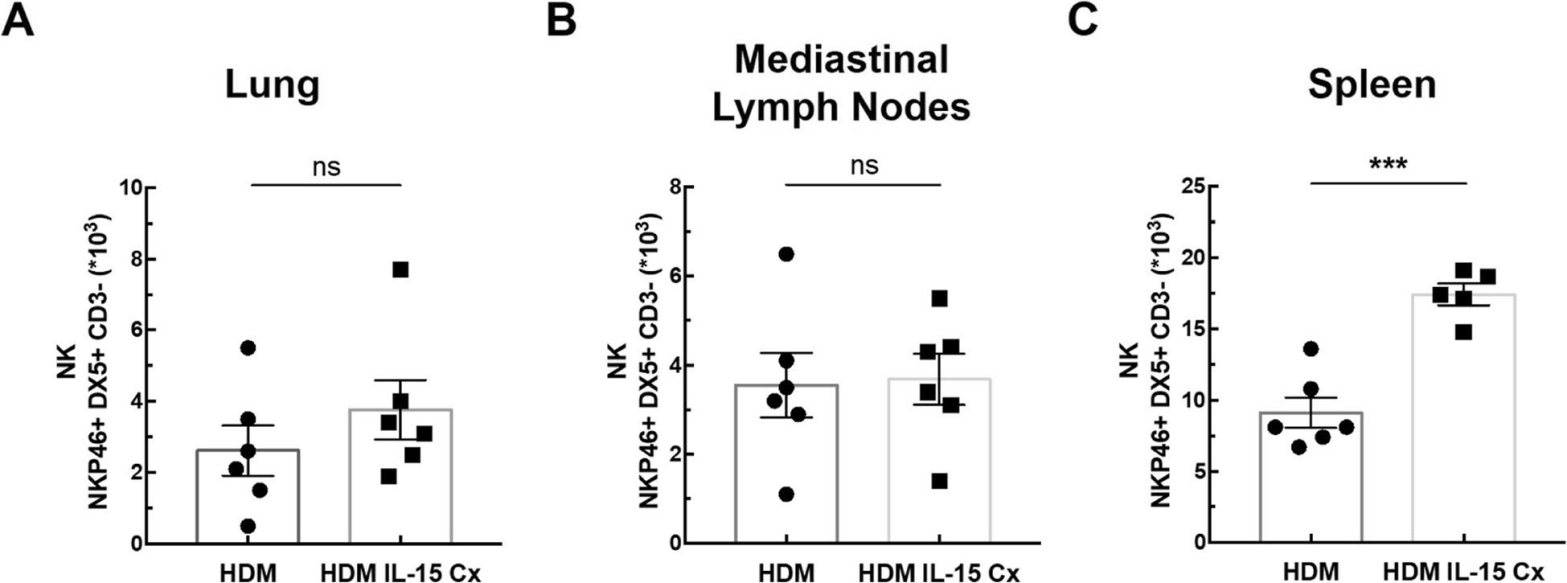
IL-15 complex increases NK cells in asthmatic mice. The numbers of NK cells (NKP46^+^, DX5^+^, CD3^−^) were respectively measured in (**a**) lungs, (**b**) mediastinal lymph nodes and (**c**) spleen by flow cytometry. The dissected lungs, mediastinal lymph nodes, and spleen from HDM and HDM IL-15 Cx were crushed, filtered, and then lysed by Red Blood Cell lysis (RBC) for lungs and spleen. The data are represented as mean ± SEM (between 5 and 6 mice per group); * *p* < 0.05, ** *p* < 0.01, *** *p* < 0,001 using parametric t-test. ns = not significant

**Fig. 6:**
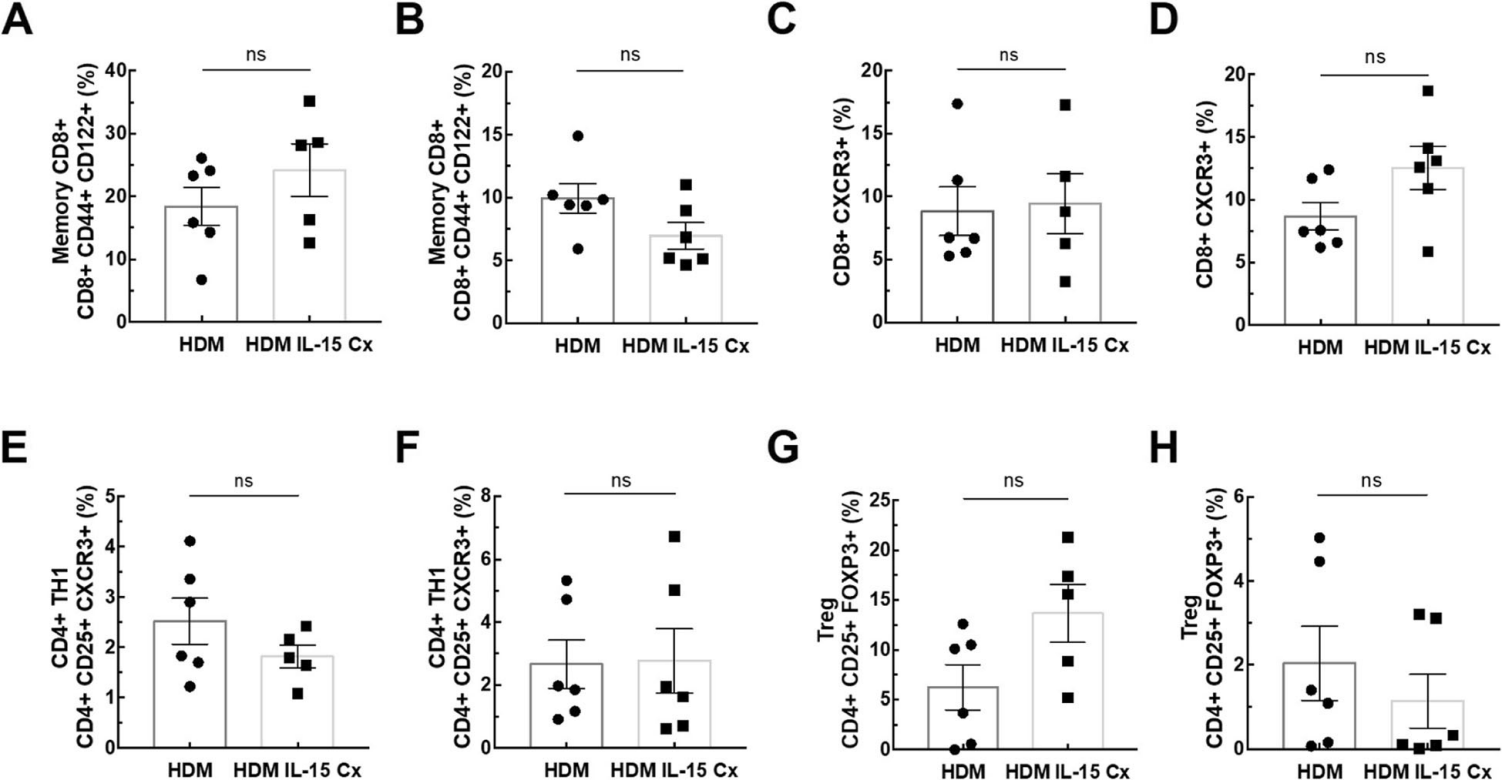
Adaptative immunity is not modified in asthmatic mice with IL-15 complex. The percentage of (**a**, **b**) Memory CD8^+^ lymphocytes (CD8^+^, CD44^+^, CD122^+^), (**c**, **d**) CD8^+^ lymphocytes secreting interferon gamma (CD8^+^, CXCR3^+^), **(e**, **f**) CD4^+^ Th1 lymphocytes (CD4^+^, CD25^+^, CXCR3^+^) and (**g**, **h**) T regulatory lymphocytes (Treg) (CD4^+^, CD25^+^, FOXP3^+^) were measured in (**a**, **c**, **e**, **g**) lungs and (**b**, **d**, **f**, **h**) spleen. The dissected lungs and spleen from HDM and HDM IL-15 Cx mice were crushed, filtered, and then lysed by RBC for lungs and spleen. The data are represented as mean ± SEM (between 5 and 6 mice per group); * *p* < 0.05 using parametric t-test. ns = not significant

Having shown that IL-15Cx has interesting properties and plays a role in modulation of NK cells, CD8^+^ lymphocytes and T regulatory lymphocytes, we aimed to evaluate effect of IL-15Cx in airway inflammation induced by house dust mite (HDM). Asthma results in chronic inflammation of the lower airways, bronchoconstriction and bronchial hypersecretion, associated with bronchial remodeling (smooth muscle hypertrophy, epithelial cell hyperplasia, basement membrane thickening). We decided to analyze these different pillars of pathophysiology with a murine model of allergic asthma to HDM **(**Fig. 4a) characterized by a mixed inflammation Th2 and Th17 [15]. We verified the levels of endogenous human and mouse IL-15 after injection of the complex in wild type mice and in asthma challenged mice. As expected, endogenous mouse IL-15 levels were not modified after IL-15Cx injection, but human IL-15 levels were increased (data not shown). An essential component for studying asthma is bronchial hyperreactivity. This can be evaluated by an invasive method that uses forced oscillometry, Flexivent®. We performed it on control group (CTL), asthmatic mice group (HDM group) and on asthmatic mice treated by IL-15Cx group (HDM IL-15Cx group) **(**Fig. 4b). As expected, asthmatic mice showed an increase in lung resistances in response to increasing doses of methacholine compared to control mice. However, there was no difference in pulmonary resistance between the asthmatic and asthmatic IL-15Cx treated mice. To better understand the lack of effect of the IL-15Cx on bronchial hyperreactivity, we performed histology on lung tissue to measure anatomical observation (Fig. 4c-d). In HDM mice, we observed a strong peri-bronchial and perivascular cell infiltrate, as well as hyperplasia of the epithelium with the appearance of numerous mucus cells (Fig. 4c). The evaluation of this histological score confirms a significant increase in the score between CTL and HDM groups (Fig. 4d). As for lung resistances, we did not show any reduction in the histological score between the HDM and HDM IL-15Cx groups. Mucus production is increased in asthma and contributes to increase pulmonary resistance. We evaluated mucus production on our model by calculating a score of goblet cells colored by periodic acid of Schiff (Fig. 4c). We found an increase in this score between CTL and HDM groups (Fig. 4d). We also observed a decrease in the score between the HDM group and the HDM IL-15Cx group. This decrease, however, seems insufficient to improve the respiratory function of our asthmatic murine model. To better assess lung inflammation in our model, we performed broncho-alveolar lavages. We found an increase in eosinophil count in our asthma model but IL-15Cx injection did not decrease eosinophilia (Fig. 4e). Then the inflammatory action of the IL-15 complex, was analyzed in lungs, mediastinal lymph nodes and spleen in HDM and HDM IL-15Cx mice. We first evaluated NK cells (NKP46^+^, DX5^+^, CD3^−^) **(**Fig. 5**)**. In lungs and in mediastinal lymph nodes we found no significant difference after the injection of the IL-15Cx (Fig. 5a-b). However, we found an increase in NK cells in the spleen between HDM and HDM IL-15Cx groups. In the allergic asthmatic murine model, IL-15 complex injected intraperitoneally has an effect on NK cells in spleen but not in local sites of inflammation. Then, we explored adaptative immunity to investigate whether the complex modulates inflammation (Fig. 6). Regarding memory CD8^+^ lymphocytes (CD8^+^, CD44^+^, CD122^+^), there was no statistical difference in the lungs and in the spleen (Fig. 6a-b) between groups. The percentage of CD8^+^ CXCR3^+^ lymphocytes was not increased in the lungs after the injection of the complex **(**Fig. 6c) but it tended to increase in the spleen (8,66% versus 12,54% respectively, *p* = 0,07) **(**Fig. 6d). Moreover, the percentage of Th1 lymphocytes (CD4^+^, CD25^+^, CXCR3^+^) was not modified in the lungs (Fig. 6e) nor in the spleen (Fig. 6f). T regulatory lymphocytes (CD4^+^, CD25^+^, FOXP3^+^) were not modified in the spleen (Fig. 6h) but tended to increase in the lungs (6,24% versus 3,67% respectively, p = 0,07) **(**Fig. 6g). In conclusion, we did not find any impact on the adaptative immunity with the IL-15 complex in our model of allergic asthma.

## Discussion

In this study, we have evaluated the therapeutic effect of an IL-15 / sIL-15Rα complex on pulmonary inflammation and in particular through a mouse model of acute HDM allergic asthma. We found that the IL-15Cx can modulate lung inflammation but has no effect on asthma features in mouse. Indeed, focusing on the three main physiopathological pillars of asthma, our results show that the IL-15Cx did not decrease bronchial hyperreactivity, did not reduce bronchial remodeling or cellular bronchial infiltrate, but reduced bronchial mucus secretion and modulated systemic inflammatory response. Using ovalbumin sensitized transgenic mice with an increased number of memory-like CD8^+^ T cells in peripheral lymphoid tissues, Th2-type cytokine production was found to be severely attenuated after inhalation of ovalbumin. Transgenic mice overexpressing IL-15 preferentially developed CD8^+^ T cell-mediated Th1 responses after ovalbumin sensitization [17]. Mathias et al. have made it possible to better characterize the role of this cytokine in the inflammatory response during the development of an allergic airway disease by using interleukin-15 deficient mice [18]. They used wild-type and IL-15 deficient mice that were sensitized and then exposed to ovalbumin. In the absence of IL-15, ovalbumin-sensitive mice exhibited increased bronchial eosinophil inflammation, high IL-13 production, and severe pulmonary histopathology damages compared to wild type mice. These results clearly demonstrate that mice with endogenous IL-15 deficiency are likely to develop a strong, potent and Th2-mediated allergic airway disease, which could be regulated by CD8^+^ T cells. Recently it has been shown that IL-15 deficiency increases airway resistance and decreases compliance in a mouse model of asthma [14]. These discrepancies with our observation might be linked to several points. First, we used a lower dose of IL-15 than others. Therefore, an insufficient dose injected could explain the absence of an effect on bronchial hyperreactivity. It is of note that the dose we used demonstrated a biological effect stronger than IL-15 alone [19]. Secondly, we used a mouse model of asthma induced by HDM mimicking the human situation in a more complex manner than the airway inflammation induced by ovalbumin. In fact, our model displays a mix Th2/Th17 inflammation associated with a mix eosinophil/neutrophil infiltrate. Therefore, it is likely that in our model, mechanisms implicated might be more complicated, yet closer to the human situation, and several compensatory effects may explain our observation. Despite the potential effect of IL-15Cx on Th2 inflammation, we did not use it on an ovalbumin-induced asthma model, a pure Th2 model. Indeed, if the development of new therapies in asthma remains essential, the last years have seen the emergence of biotherapies efficiently targeting the Th2 pathway (anti-IL5, anti-RIL-5) in severe asthma [20, 21]. Research must therefore now focus on patients with a part of non-Th2 inflammation in which oral corticosteroids is often the only option, with unacceptable side effects. We therefore went directly on a mouse model with a mixed Th2 and non-Th2 inflammation, which has already proven itself [15]. After the study of bronchial hyperreactivity we were interested in the action of the complex on bronchial remodeling and cellular infiltrate. The complex does not seem to interfere with bronchial remodeling. Nevertheless, the analysis of the secretion of mucus by bronchial goblet cells through the use of a score after PAS staining, found a significant decrease thereof. The mechanism of action explaining this effect remains to be elucidated by studying the components of the IL-15 receptor present on the goblet cells of asthmatic subjects. With respect to inflammatory modulation, we observed that in control mice, injection of the IL-15 / sIL-15Rα complex had systemic and a local effect on NK cells. In addition, we observed an increase in CD8^+^ memory and T regulatory lymphocytes. NK cells have an immunoregulatory role in many inflammatory pathologies and their involvement in asthma remains highly controversial in the literature. The development of house dust mite allergic asthma in mice has been shown to increase NK, particularly in the mediastinal lymph nodes, but NK blockade by antibodies or their genetic deficiency does not alter the characteristics of asthma, namely Th2 inflammation, bronchial hyperreactivity, specific IgE titers, and mucus production [22]. The study of the prostaglandin I2 receptor deficiency in a HDM allergic murine model shows an increase in IFN-γ producing NK cells in the lungs, inversely correlated with the number of type 2 innate lymphoid cells (ILC2). In addition, anti-NK-1.1 monoclonal antibody treatment restored inflammation of the allergic airways and increased the level of IL-5 producing, attracting eosinophils. This study thus reveals that NK cells prevent allergic pulmonary inflammation by limiting the number of ILC2 [23]. Furthermore, the mode of administration of the complex can influence the biological activity and explain our results showing a difference in the spleen (systemic) and not at the tissue level. In this context, the administration of the complex by aerosol would be an interesting track. Finally, viral exacerbations are the leading cause of exacerbation of asthma in winter, and an increase in memory T8 lymphocytes would perhaps reduce these exacerbations in targeted populations. Indeed, Laza-Stanca et al demonstrated that IL-15 deficiency in humans could be a part of the virus-induced asthma exacerbations pathogenesis [24]. In asthma, Th1 and Treg populations are not directly involved in the allergic reaction but are rather involved as antagonists and regulators [25]. Th1 population, secreting interferon gamma (IFNγ), will modulate the allergic reaction by limiting the expansion of the Th2 population and inducing cell-mediated immunity [26]. The other regulatory T cell population, via the secretion of IL-10 and tumor growth factor beta (TGF-β), will inhibit the Th1, Th2 and Th17 responses to restore the pro-inflammatory/anti-inflammatory balance [27]. Tregs inactivate the inflammatory response in order to stop bronchial inflammation but are quantitatively and qualitatively deficient in this pathology [28, 29]. In our model, the increase in Th1 CXCR3^+^ in the Th1 pathway may also counteract the Th2 inflammation but these modulations do not seem to be sufficient. In the same way, a tendency to increase regulatory T cells in the lungs after injection of the complex is observed. Though we did not demonstrate a therapeutic effect of the IL-15Cx in our mouse model of house dust mite-allergic asthma, other therapies implicating IL-15 in asthma should not be completely abandoned. Indeed, it is clearly established that asthma syndrome recovers several different phenotypes with different endotypes, so that targeting one molecule could be relevant for one endotype but not for another. For instance, targeting IL-5 with mepolizumab on benralizumab is relevant for the eosinophilic phenotype whereas omalizumab concerns allergic asthma. IL-15 could be involved in another type of asthma for which our model, mainly Th2 and Th17-driven, is not relevant. Nevertheless, the modulation of inflammation induced by the IL-15 / sIL-15Rα complex is of major interest in inflammatory diseases such as psoriasis, transplantation or in oncology where the role of IL-15 is well established. Indeed, we demonstrated before that endogenous soluble IL-15Rα derived from epidermal stroma, protects against dendritic cell/IL-15-mediated, T cell-driven skin inflammation in vivo, and is relevant to human psoriasis [30]. Regarding tolerance in lung transplantation, *Jungraithmayr* et al. showed that expansion of recipient NK cells through IL-15/IL-15Rα complex treatment resulted in decreased T-cell infiltration and alloreactive T-cell priming as well as improved function of the allogeneic lung transplant in a mouse model [31]. Other forms of the complex have been tested in oncology. A fusion protein called RLI, composed of the sushi domain of IL-15 receptor α coupled via a linker to IL-15, seems to have a high antitumor activity in metastatic melanoma and colorectal cancer in mice [32]. ALT-803, is a new IL-15 superagonist composed of a mutant of human IL-15 and the sushi domain of human IL-15 Rα fused with the Fc domain IgG1. Recently, the use of ALT-803 in combination with NIVOLUMAB (anti-PD1) in non-small-cell metastatic lung cancers has been the subject of an encouraging Phase 1 study that has conducted to a Phase 2 study still in progress [33]. A novel fusion protein encompassing anti-PD-L1 and the IL-15 superagonist fusion complex (ALT-803) called N-809 has been created and has very promising antitumor functionalities [34]. The association of the complex with other existing targeted therapies could therefore also be an interesting research avenue**.**

## Conclusions

In conclusion, we did not demonstrate an effect of the IL-15 / sIL-15Rα complex in our mouse model of HDM-allergic asthma on bronchial hyperreactivity, bronchial remodeling and inflammatory response. A decrease of mucus secretion has been observed but needs further explanation. On the other hand, we showed a modulation of lung and systemic inflammation with an increase of NK cells, CD8^+^ memory and CD8^+^ CXCR3^+^ lymphocytes in control mice. This could be of interest for other inflammatory disease associated or not with other biologics. Indeed, the addition of therapies could potentiate the inflammatory modulation or act on other inflammatory pathways.

## Data Availability

The datasets used and/or analyzed during the current study are available from the corresponding author on reasonable request.

## Abbreviations

HDM: House dust mite
IL-15 Rα: Alpha chain receptor of IL-15
IL-15Cx: IL-15/ sIL-15R ***α*** complex
NK: Natural killer cells
Treg: T regulatory lymphocytes

## References

[1] Burton JD, Bamford RN, Peters C, Grant AJ, Kurys G, Goldman CK, Brennan J, Roessler E, Waldmann TA (1994). A lymphokine, provisionally designated interleukin T and produced by a human adult T-cell leukemia line, stimulates T-cell proliferation and the induction of lymphokine-activated killer cells. Proc Natl Acad Sci U S A.

[2] Giri JG, Ahdieh M, Eisenman J, Shanebeck K, Grabstein K, Kumaki S, Namen A, Park LS, Cosman D, Anderson D (1994). Utilization of the beta and gamma chains of the IL-2 receptor by the novel cytokine IL-15. EMBO J.

[3] Anderson DM, Kumaki S, Ahdieh M, Bertles J, Tometsko M, Loomis A, Giri J, Copeland NG, Gilbert DJ, Jenkins NA (1995). Functional characterization of the human interleukin-15 receptor alpha chain and close linkage of IL15RA and IL2RA genes. J Biol Chem.

[4] Kennedy MK, Glaccum M, Brown SN, Butz EA, Viney JL, Embers M, Matsuki N, Charrier K, Sedger L, Willis CR (2000). Reversible defects in natural killer and memory CD8 T cell lineages in interleukin 15-deficient mice. J Exp Med.

[5] Dubois S, Patel HJ, Zhang M, Waldmann TA, Muller JR (2008). Preassociation of IL-15 with IL-15R alpha-IgG1-fc enhances its activity on proliferation of NK and CD8+/CD44high T cells and its antitumor action. J Immunol.

[6] Zhang X, Sun S, Hwang I, Tough DF, Sprent J (1998). Potent and selective stimulation of memory-phenotype CD8+ T cells in vivo by IL-15. Immunity.

[7] Dubois S, Mariner J, Waldmann TA, Tagaya Y (2002). IL-15Ralpha recycles and presents IL-15 in trans to neighboring cells. Immunity.

[8] Sandau MM, Schluns KS, Lefrancois L, Jameson SC (2004). Cutting edge: transpresentation of IL-15 by bone marrow-derived cells necessitates expression of IL-15 and IL-15R alpha by the same cells. J Immunol.

[9] Mortier E, Bernard J, Plet A, Jacques Y (2004). Natural, proteolytic release of a soluble form of human IL-15 receptor alpha-chain that behaves as a specific, high affinity IL-15 antagonist. J Immunol.

[10] Mortier E, Quemener A, Vusio P, Lorenzen I, Boublik Y, Grotzinger J, Plet A, Jacques Y (2006). Soluble interleukin-15 receptor alpha (IL-15R alpha)-sushi as a selective and potent agonist of IL-15 action through IL-15R beta/gamma. Hyperagonist IL-15 x IL-15R alpha fusion proteins. J Biol Chem.

[11] Rubinstein MP, Kovar M, Purton JF, Cho JH, Boyman O, Surh CD, Sprent J (2006). Converting IL-15 to a superagonist by binding to soluble IL-15R{alpha}. Proc Natl Acad Sci U S A.

[12] Bouchaud G, Garrigue-Antar L, Sole V, Quemener A, Boublik Y, Mortier E, Perdreau H, Jacques Y, Plet A (2008). The exon-3-encoded domain of IL-15ralpha contributes to IL-15 high-affinity binding and is crucial for the IL-15 antagonistic effect of soluble IL-15Ralpha. J Mol Biol.

[13] Stoklasek TA, Schluns KS, Lefrancois L (2006). Combined IL-15/IL-15Ralpha immunotherapy maximizes IL-15 activity in vivo. J Immunol.

[14] Venkateshaiah SU, Zhu X, Rajavelu P, Niranjan R, Manohar M, Verma AK, Lasky JA, Mishra A (2018). Regulatory effects of IL-15 on allergen-induced airway obstruction. J Allergy Clin Immunol.

[15] Chesne J, Braza F, Chadeuf G, Mahay G, Cheminant MA, Loy J, Brouard S, Sauzeau V, Loirand G, Magnan A (2015). Prime role of IL-17A in neutrophilia and airway smooth muscle contraction in a house dust mite-induced allergic asthma model. J Allergy Clin Immunol.

[16] Ezeamuzie CI, El-Hashim AZ, Renno WM, Edafiogho IO (2014). Antiallergic and antiasthmatic effects of a novel enhydrazinone ester (CEE-1): inhibition of activation of both mast cells and eosinophils. J Pharmacol Exp Ther.

[17] Ishimitsu R, Nishimura H, Yajima T, Watase T, Kawauchi H, Yoshikai Y (2001). Overexpression of IL-15 in vivo enhances Tc1 response, which inhibits allergic inflammation in a murine model of asthma. J Immunol.

[18] Mathias CB, Schramm CM, Guernsey LA, Wu CA, Polukort SH, Rovatti J, Ser-Dolansky J, Secor E, Schneider SS, Thrall RS, Aguila HL (2017). IL-15-deficient mice develop enhanced allergic responses to airway allergen exposure. Clin Exp Allergy.

[19] Elpek KG, Rubinstein MP, Bellemare-Pelletier A, Goldrath AW, Turley SJ (2010). Mature natural killer cells with phenotypic and functional alterations accumulate upon sustained stimulation with IL-15/IL-15Ralpha complexes. Proc Natl Acad Sci U S A.

[20] Busse P, Wechsler ME (2018). Identification of remission in adult-onset asthma. Lancet Respir Med.

[21] Ortega HG, Liu MC, Pavord ID, Brusselle GG, FitzGerald JM, Chetta A, Humbert M, Katz LE, Keene ON, Yancey SW (2014). Mepolizumab treatment in patients with severe eosinophilic asthma. N Engl J Med.

[22] Haspeslagh E, van Helden MJ, Deswarte K, De Prijck S, van Moorleghem J, Boon L, Hammad H, Vivier E, Lambrecht BN. Role of NKp46(+) natural killer cells in house dust mite-driven asthma. EMBO Mol Med. 2018;10(4):e8657. 10.15252/emmm.201708657 PMC5887908 29444897

[23] Simons B, Ferrini ME, Carvalho S, Bassett DJ, Jaffar Z, Roberts K (2017). PGI2 controls pulmonary NK cells that prevent airway sensitization to house dust mite allergen. J Immunol.

[24] Laza-Stanca V, Message SD, Edwards MR, Parker HL, Zdrenghea MT, Kebadze T, Kon OM, Mallia P, Stanciu LA, Johnston SL (2011). The role of IL-15 deficiency in the pathogenesis of virus-induced asthma exacerbations. PLoS Pathog.

[25] Magnan AO, Mely LG, Camilla CA, Badier MM, Montero-Julian FA, Guillot CM, Casano BB, Prato SJ, Fert V, Bongrand P, Vervloet D (2000). Assessment of the Th1/Th2 paradigm in whole blood in atopy and asthma. Increased IFN-gamma-producing CD8(+) T cells in asthma. Am J Respir Crit Care Med.

[26] Manetti R, Parronchi P, Giudizi MG, Piccinni MP, Maggi E, Trinchieri G, Romagnani S (1993). Natural killer cell stimulatory factor (interleukin 12 [IL-12]) induces T helper type 1 (Th1)-specific immune responses and inhibits the development of IL-4-producing Th cells. J Exp Med.

[27] Haribhai D, Williams JB, Jia S, Nickerson D, Schmitt EG, Edwards B, Ziegelbauer J, Yassai M, Li SH, Relland LM (2011). A requisite role for induced regulatory T cells in tolerance based on expanding antigen receptor diversity. Immunity.

[28] Lloyd CM, Hawrylowicz CM (2009). Regulatory T cells in asthma. Immunity.

[29] Zhao ST, Wang CZ (2018). Regulatory T cells and asthma. J Zhejiang Univ Sci B.

[30] Bouchaud G, Gehrke S, Krieg C, Kolios A, Hafner J, Navarini AA, French LE, Boyman O (2013). Epidermal IL-15Ralpha acts as an endogenous antagonist of psoriasiform inflammation in mouse and man. J Exp Med.

[31] Jungraithmayr W, Codarri L, Bouchaud G, Krieg C, Boyman O, Gyulveszi G, Becher B, Weder W, Munz C (2013). Cytokine complex-expanded natural killer cells improve allogeneic lung transplant function via depletion of donor dendritic cells. Am J Respir Crit Care Med.

[32] Bessard A, Sole V, Bouchaud G, Quemener A, Jacques Y (2009). High antitumor activity of RLI, an interleukin-15 (IL-15)-IL-15 receptor alpha fusion protein, in metastatic melanoma and colorectal cancer. Mol Cancer Ther.

[33] Wrangle JM, Velcheti V, Patel MR, Garrett-Mayer E, Hill EG, Ravenel JG, Miller JS, Farhad M, Anderton K, Lindsey K (2018). ALT-803, an IL-15 superagonist, in combination with nivolumab in patients with metastatic non-small cell lung cancer: a non-randomised, open-label, phase 1b trial. Lancet Oncol.

[34] Fujii R, Jochems C, Tritsch SR, Wong HC, Schlom J, Hodge JW (2018). An IL-15 superagonist/IL-15Ralpha fusion complex protects and rescues NK cell-cytotoxic function from TGF-beta1-mediated immunosuppression. Cancer Immunol Immunother.

